# Changes in balance and joint position sense during a 12-day high altitude trek: The British Services Dhaulagiri medical research expedition

**DOI:** 10.1371/journal.pone.0190919

**Published:** 2018-01-17

**Authors:** Sarah B. Clarke, Kevin Deighton, Caroline Newman, Gareth Nicholson, Liam Gallagher, Christopher J. Boos, Adrian Mellor, David R. Woods, John P. O’Hara

**Affiliations:** 1 Institute for Sport, Physical Activity & Leisure, Leeds Beckett University, Leeds, United Kingdom; 2 School of Health and Human Performance, Northern Michigan University, Marquette, Michigan, United States of America; 3 Defence Medical Services, Lichfield, United Kingdom; 4 Department of Cardiology, Poole Hospital NHS Trust, Longfleet Rd, Poole, United Kingdom; 5 Department of Postgraduate Medical Education, Bournemouth University, Bournemouth, United Kingdom; 6 South Tees Hospitals NHS Trust, James Cook University Hospital, Middlesbrough, United Kingdom; 7 Northumbria and Newcastle NHS Trusts, Wansbeck General and Royal Victoria Infirmary, Newcastle, United Kingdom; 8 University of Newcastle, Newcastle upon Tyne, United Kingdom; National Yang-Ming University, TAIWAN

## Abstract

Postural control and joint position sense are essential for safely undertaking leisure and professional activities, particularly at high altitude. We tested whether exposure to a 12-day trek with a gradual ascent to high altitude impairs postural control and joint position sense. This was a repeated measures observational study of 12 military service personnel (28±4 years). Postural control (sway velocity measured by a portable force platform) during standing balance, a Sharpened Romberg Test and knee joint position sense were measured, in England (113m elevation) and at 3 research camps (3619m, 4600m and 5140m) on a 12-day high altitude trek in the Dhaulagiri region of Nepal. Pulse oximetry, and Lake Louise scores were also recorded on the morning and evening of each trek day. Data were compared between altitudes and relationships between pulse oximetry, Lake Louise score, and sway velocity were explored. Total sway velocity during standing balance with eyes open (p = 0.003, d = 1.9) and during Sharpened Romberg test with eyes open (p = 0.007, d = 1.6) was significantly greater at altitudes of 3619m and 5140m when compared with sea level. Anterior-posterior sway velocity during standing balance with eyes open was also significantly greater at altitudes of 3619m and 5140m when compared with sea level (p = 0.001, d = 1.9). Knee joint position sense was not altered at higher altitudes. There were no significant correlations between Lake Louise scores, pulse oximetry and postural sway. Despite a gradual ascent profile, exposure to 3619 m was associated with impairments in postural control without impairment in knee joint position sense. Importantly, these impairments did not worsen at higher altitudes of 4600 m or 5140 m. The present findings should be considered during future trekking expeditions when developing training strategies targeted to manage impairments in postural control that occur with increasing altitude.

## Introduction

Travel to high altitude among lowlanders has become increasingly common for both professional and leisure purposes. Trekking in Nepal has become one of these popular high altitude adventurous activities; the number of trekking permits obtained by foreigners rose from 14000 in 1985 [1] to 97185 in 2014 [2]. Acute and chronic exposure to high altitude environments may result in clinical syndromes ranging from high altitude headache to acute mountain sickness (AMS) and high altitude cerebral edema (HACE). It has been suggested that these syndromes manifest along a spectrum transitioning from AMS to HACE with the presentation of serious neurological impairments such as balance and postural control [3]. Maintaining balance and posture is a function of a number of sensory inputs to the central nervous system that include visual, vestibular, and somatosensory components [4]. Postural control is an essential factor required to perform many daily activities and has been utilised as a factor to classify elderly fallers and non-fallers [5]. Travelling over dangerous mountainous terrain has its own inherent risks, and compounding this with these impairments in balance and postural control makes the trekker particularly vulnerable to trips or falls [5].

Decrements in balance that may lead to trips or falls have been well documented during short exposures of 24 hours [6] or less (<60 mins [7–10]) to simulated hypobaric and normobaric hypoxia. Decrements occur at both high (1500 m to 3500m) and very high altitudes (3500m to 5500m) [6–10], and were also reported during prolonged exposure over two days at terrestrial altitudes of 1630 m and 2590 m [11]. Decrements at terrestrial altitude have been shown to persist for three days at altitudes of 4559 m [12]. Although balance was demonstrated to be impaired at high [12] and very high [6] altitude, few studies have shown any association between balance and AMS. It remains unclear whether such decrements persist or are magnified during more prolonged gradual ascents at these altitudes exceeding four days, such as would be experienced during a high altitude trek where acclimatisation occurs in response to the increasing altitude. Therefore, further field research on expedition trekking above 5000 m is warranted, where the occurrence of AMS and HACE is significantly more likely to manifest [3]. Decrements in balance and postural control are especially important, may be magnified, and could provide stronger diagnostic links with AMS and HACE syndromes.

The control of static and dynamic posture involves inputs from a number of sensory inputs to the central nervous system. Although the visual and vestibular inputs have been accounted for in previous assessments of balance at high altitude [6,8,10–12], somatosensory inputs have received limited attention. Assessments of balance using a moving platform indicated some impairment in somatosensory input at simulated hypoxia [7,13]. Performance in this task was impaired at 1524 m [7] and 5000 m [13] but the exact sensory mechanisms responsible remain unclear. The adaptation of the somatosensory system in extreme cold environments has been investigated using joint position sense [14]. Since a measurement of joint position sense acts as an isolated investigation of the somatosensory system, measurement of joint position sense at high altitudes provides a clearer picture regarding the mechanisms by which decrements in balance occur at these high altitudes.

Therefore the purpose of the current study was to investigate balance and knee joint position sense in healthy participants at sea-level, 3619 m, 4600 m, and 5140 m during a 12-day trek in the Dhaulagiri region of Nepal.

## Materials and methods

### Design of the study

**Fig 1:**
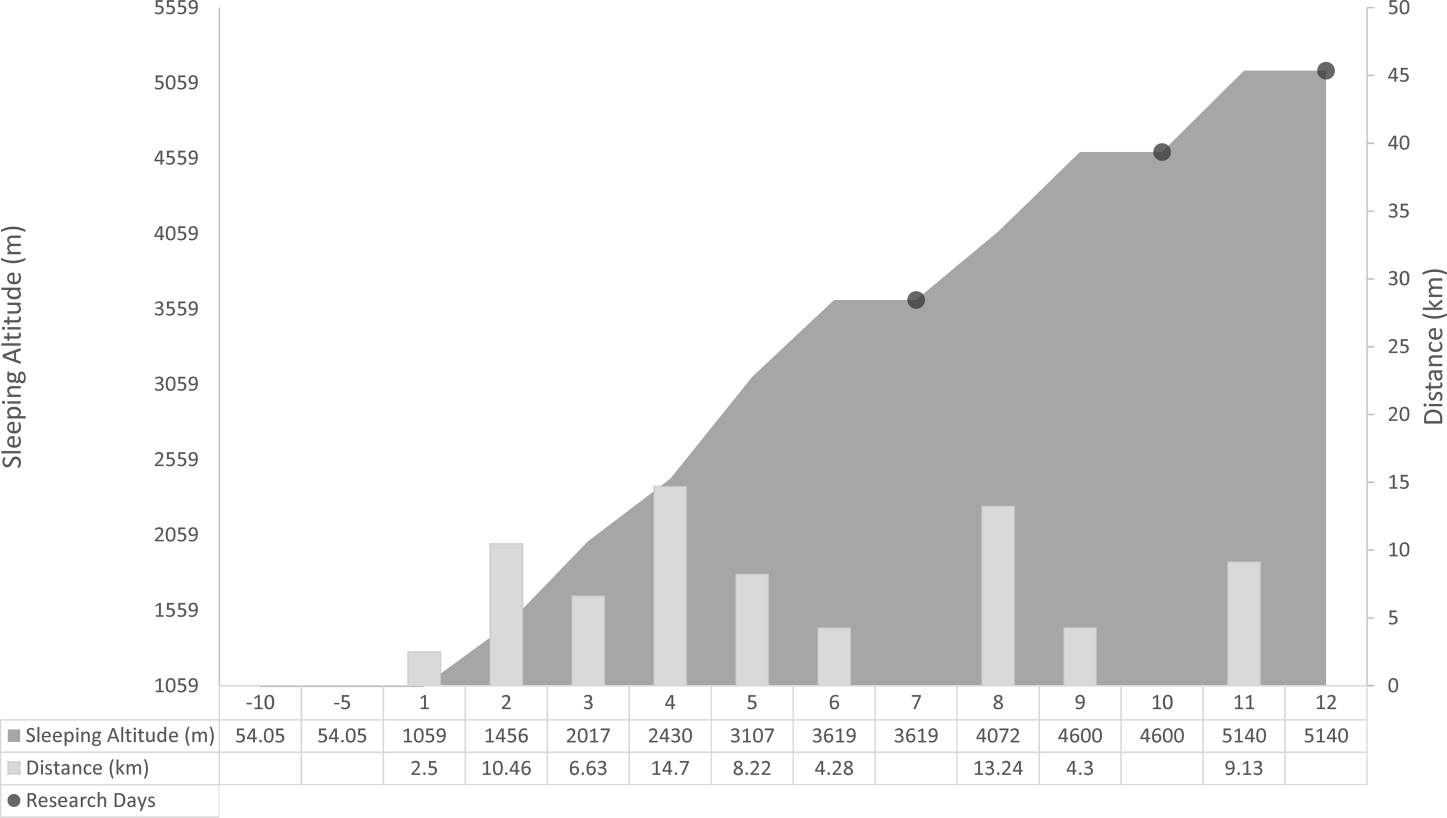
Trek itinerary and altitude profile. Daily trekking distance and sleeping altitude profile for participants utilized in this research.

This study represents part of the ‘British Services Dhaulagiri Medical Research Expedition’, which took place in March–May 2016 (for a comprehensive overview of the expedition protocol, please see Mellor et al. (2017) [15]). In April 2016, participants travelled from England to Nepal and completed a 14-day trek around the Dhaulagiri circuit in the Himalayas. Travel from England to Nepal lasted for one day, and participants were in Nepal for three days prior to starting the trek. The trek commenced from Darbang (~1059 m), peaked on day 11 at the ‘French Pass’ (~5300m) and ended on day 14 at Marpha (~2717m). Measurements were performed, in the period from March to April 2016, during one day at Leeds Beckett University, Leeds, UK (Sea level [SL]: 113 m), and at research days at each fixed camp, during a 12-day portion of the trek in the Dhaulagiri region of Nepal: One day at Italian Base Camp (IBC: Trek day 7; 3619 m), one day at Dhaulagiri Base Camp (DBC: Trek Day 10; 4600 m), and one day at Hidden Valley (HV: Trek Day 12; 5140 m). Details of the trek itinerary and sleeping altitude are illustrated in Fig 1. On the days preceding data collection at IBC, DBC, and HV participants walked 4.3, 4.3, and 9.1 km and gained an elevation of 512, 528, and 540 m, respectively. All trekking on these days was completed by 5 pm. Following an overnight rest, participants avoided strenuous exercise and stayed within 200 m of the study location on research days. No participants were treated with prophylactic acetazolamide (Diamox®), and no other medications were used for AMS or which would have interfered with balance or the central nervous system.

### Participants

Twelve healthy British military service personnel (9 males and 3 females), mean ± SD: aged 28 ± 4 years; body mass 71.3 ± 10.3 kg; body mass index (BMI) 23.0 ± 2.1 kg m^−2^ participating in a military Adventurous Training exercise to the Dhaulagiri region of Nepal volunteered for the current study. All volunteers were sea level residents and had not been to an altitude over 1000 m for at least 3 months. All participants were non-smokers, were confirmed as healthy by completing a detailed health questionnaire, confirmed they were not regularly taking any medications, and had no current or previous history of neurological conditions. In addition, participants were physically fit and could run 2.4 km on a treadmill at a 2% gradient in under 13 min 37 s in accordance with military requirements. The study protocol was approved by the Ministry of Defence Research Ethics Committee, and was conducted according to the standards of the Declaration of Helsinki. All participants provided written informed consent.

### Daily measures

AMS was assessed every morning and evening using the Lake Louis Scoring System (LLS) [16]. The LLS scales allocate a score of 0 (symptom not present) to 3 (severe) for symptoms of AMS (headache, gastrointestinal symptoms, fatigue/weakness, dizzy/light-headedness, difficulty sleeping). A total score of ≥3 in the presence of a headache is consistent with AMS [16]. Previous research also utilized a score of ≥6 to indicate severe or debilitating AMS [17]. Pulse Oximetry (SpO_2_) was measured via a fingertip pulse oximeter (Nellcor™ PM10N; Medtronic, Minneapolis, MN) every morning and evening while participants were resting in a seated position.

### Postural stability

Balance was quantified at sea level and at the three research camps with a portable force platform (9286B, Kistler, Winterthur, Switzerland). The force platform was levelled and stabilised within a research tent at each location using specialised levelling feet (JVD Design & Automation Ltd, Leeds, UK). Repeated calibrations with known weights were also performed on the morning of each data collection. Balance was quantified via measures of centre of pressure velocity as outlined in the data analysis section below. Standing balance was assessed standing on both legs with eyes either opened or closed for 3 trials (6 trials total). For all standing balance trials, an average of three trials was used for further statistical analysis. Measurements lasted 30 s with breaks of at least 20 s between trials [11]. Standing balance was performed with feet positioned parallel 7 cm apart and arms in the fundamental standing position during all measurements [8,10,12].

Balance was also assessed during a Sharpened Romberg Test (SRT) in which the SRT score was recorded and the portable force platform used to measure centre of pressure velocity. SRT is a commonly used field test for assessment of postural control in high altitude scenarios [18]. The SRT test procedure was in accordance with previous research [18], during which participants were asked to stand with feet heel-to-toe in a straight line, the dominant foot behind non-dominant foot. Participants then placed both arms across their chest with hands on the opposite shoulder. Once stable, participants were asked to close their eyes and maintain this position for 60 s. If the first test was successful, no further testing was required and participants assumed a maximum score of 240 seconds (60*4). A failed test occurred if the eyes were opened, arms were extended to regain posture, or feet were moved to regain balance. If a failed test occurred on the first attempt, the participant attempted up to three further trials of 60 seconds, and a sum of the times was recorded. If a test of 60 seconds was completed on the second or third trial, all subsequent tests assumed a score of 60. Participants completed a maximum of four trials with a maximum or normal SRT of 240 [18].

### Knee joint position sense

Active ipsilateral limb repositioning of the right knee was assessed after passive positioning, using two-dimensional videography of the right leg [19]. This measurement has been validated as reliable in the clinical setting and has been suggested as an appropriate test for determination of knee joint position sense (KJPS) in clinical studies [20,21]. A Camera (Casio Exilim, EX-FC100, Casio Electronics Co., Ltd. London, UK; 30 Hz) placed on a fixed level tripod 3 m perpendicular to the knee motion recorded the motion of the knee throughout the procedure.

The participants wore close fitting shorts or leggings and were prepared for data collection by placing markers on the greater trochanter, the lateral epicondyle, and the lateral malleolus of the right leg. Participants were seated in a position where the leg was flexed at approximately 90° and the popliteal fossa was not in contact with the edge of the seat [14]. It was not possible to control the environmental temperature during this field based research, however the literature has reported no decrements in KJPS [14] or postural stability [22] following reductions in body temperature. To reduce the contribution of visual input, participants were blindfolded during the testing procedures. The limb was then extended by the examiner at a slow steady speed (~10°/s) to an index angle between 10°and 30°, or 30° and 60° of flexion. The participants were asked to hold this position for ~ 5 seconds [14]; the examiner then returned the leg to its starting position at the same joint angular velocity. Participants were then asked to actively reproduce the predetermined index angle with the same limb three times. In order to allow the participants to refocus after each trial, the participants left the chair and briefly walked around the laboratory or research tent.

### Data analysis

Specialised software (MARS, Kistler, Winterthur, Switzerland) was used to assess the movements of the centre of pressure (CoP), calculating the following balance parameters during 30 s of stance: average total CoP velocity (CoPV), average CoP movement velocity in anterior posterior direction (CoPVa-p), average CoP movement velocity in medial lateral direction (CoPVm-l). For each parameter during all standing balance trials, an average of three trials was used for further statistical analysis. As three trials were not always required for SRT tests, the final SRT trial for either the eyes open or eyes closed condition were used for analysis. CoP velocity constitutes a good index of the activity required to maintain stability and has been used in previous investigations at altitude [12,23]. CoP velocity is considered as a sensitive and discriminant variable of postural stability [24].

Knee angles were measured using Kinovea software (v 0.8.15, Kinovea, www.kinovea.com). For each KJPS trial, the actual error was calculated by subtracting the reproduced angle from the index angle. Positive and negative angles represented an overestimation or underestimation respectively. Absolute mean error (the average error in the three trials ignoring the direction of the error), relative error (the average of the errors in the three trials taking into account the direction of the error), and variable error (the standard deviation of the three relative error measurements) were calculated [14].

### Statistical methods and power calculations

Data were analysed using IBM SPSS® Statistics (v 24, IBM, New York, USA). The Kolmogorov-Smirnov test and inspection of the data was undertaken to assess normality of balance and KJPS data. All data are presented as mean ± standard deviations. Changes between altitudes for balance, KJPS, SpO_2_ and SRT Score data were assessed using a One-Way Repeated Measures ANOVA with Bonferroni-adjusted *post-hoc* t-tests and effect size (Cohen’s d). The correlation between LLS Score, SpO_2_ and postural sway variables were examined using Pearson’s correlation coefficients. A Type I error rate of 0.05 was maintained throughout the statistical evaluation. The scale for classification of effect size was based on Cohen [25] with 0.2, 0.5, and 0.8 representing *small*, *moderate*, and *large* differences respectively. Priority was placed on large effect sizes where the percent of non-overlap between data sets is ≥ 47.7% [25].

The sample size of 12 participants was deemed adequate to determine a significant effect for postural sway outcomes based on previously reported data which was collected during a 3-day sojourn at 4559 m [12]. This calculation was performed using G*Power 3 [26] with an alpha value of 5% and a power of 80%.

## Results

**Fig 2:**
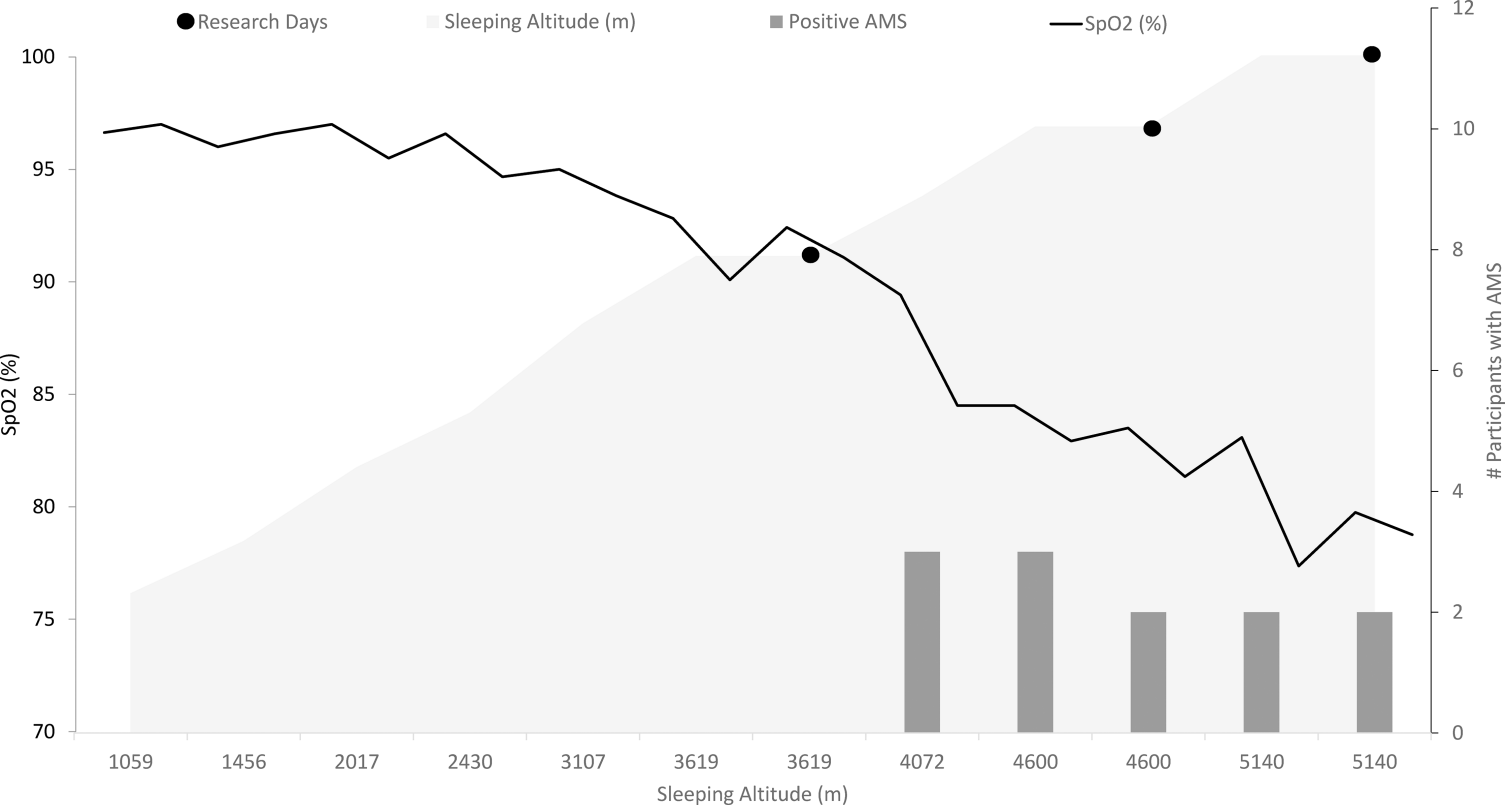
Occurrence of AMS and SpO_2_ (%) during the trek. The number of participant’s classed by the LLS as having AMS are illustrated along with changes in SpO_2_ during all days of the trek.

Changes in the occurrence of AMS and SpO_2_ with increases in trekking altitude are illustrated in Fig 2. SpO_2_ decreased significantly between trek day one and each of the three research altitudes (p<0.001) (S1 Table). Six of the twelve participants had positive LLS scores at some point on the trek. No positive scores were recorded in the period prior to IBC. Four participants reported positive scores in the period between IBC and DBC. Five participants reported positive scores in the period between DBC and HV (refer to S2 Table).

Table 1 and Table 2 summarize the COPV and COPa-p at the three research altitudes. S3–S8 Tables show corresponding values for COPm-l, KJPS, and SRT Score. The COPV during standing balance and during SRT was significantly greater at altitudes of 3619m (Standing Balance: p = 0.006, d = 1.4; SRT: p = 0.003, d = 1.9) and 5140 m (Standing Balance: p = 0.007, d = 1.6; SRT: p = 0.021 d = 1.1) compared to sea level with eyes open (Table 1). There were no significant differences for COPV with eyes closed but effect sizes were *large*, demonstrating greater COPV during standing balance at all three altitudes compared to sea level (Table 1).

**Table 1:** Centre of pressure velocity at different altitudes.

Measurement	Eyes	Sea Level	IBC 3619 m	DBC 4600 m	HV 5140 m	P ANOVA Overall
**Standing Balance**	Open	8.54 ± 1.45	11.42 ± 2.61*^¶^	11.84 ± 3.54	11.78 ± 2.62*^¶^	**0.033**
**Standing Balance**	Closed	11.32 ± 3.01	13.17 ± 3.30^**¶**^	15.22 ± 5.29^**¶**^	13.22 ± 3.28^**¶**^	**0.020**
**SRT**	Open	27.19 ± 11.57	49.94 ± 18.49*^¶^	36.06 ± 9.21	40.95 ± 12.67*^¶^	**0.007**
**SRT**	Closed	62.49 ± 26.31	63.49 ± 14.36	80.36 ± 26.27	80.30 ± 32.28	0.140

Data are presented as mean COPV in cm/s ± standard deviation. P ANOVA overall: Repeated Measures ANOVA within subject effects (SL, IBC, DBC, HV). (**Bold** < 0.05)

* p<0.05 compared with sea level

^¶^ Cohen’s d > 0.8 compared with sea level

**Table 2:** Centre of pressure velocity in the anterior-posterior direction at different altitudes.

Measurement	Eyes	Sea Level	IBC 3619 m	DBC 4600 m	HV 5140 m	P ANOVA Overall
**Standing Balance**	Open	5.90 ± 1.28	8.87 ± 1.92*^¶^	9.41 ± 2.98	9.42 ± 2.37*^¶^	**0.001**
**Standing Balance**	Closed	8.20 ± 2.75	10.53 ± 2.92^**¶**^	12.96 ± 4.97^**¶**^	10.55 ± 3.01^**¶**^	0.471
**SRT**	Open	17.29 ± 8.05	36.79 ± 14.90^**¶**^	27.05 ± 7.86^**¶**^^**#**^	29.99 ± 10.20^**¶**^	0.681
**SRT**	Closed	41.63 ± 14.15	46.90 ± 11.56	59.16 ± 17.44	58.34 ± 17.30^**¶**^	0.297

Data are presented as mean COPV in cm/s ± standard deviation. P ANOVA overall: Repeated Measures ANOVA within subject effects (SL, IBC, DBC, HV). (Bold < 0.05)

* p<0.05 compared with sea level

^¶^ Cohen’s d > 0.8 compared with sea level

^#^ Cohen’s d > 0.8 compared with IBC

The COPVa-p during standing balance with eyes open was significantly greater at altitudes of 3619m (p = 0.003, d = 1.9) and 5140 m (p = 0.001, d = 1.9) with *large* effect sizes compared with sea level (Table 2). There were no significant differences for COPVa-p when eyes were closed, but effect sizes were *large*, demonstrating greater COPVa-p during standing balance for all three altitudes compared with sea level. There were no significant differences for SRT with eyes open or closed. However, with eyes open, *large* effect sizes demonstrated a greater COPVa-p at all three altitudes compared with sea level. In contrast, during SRT with eyes closed, a *large* effect size only demonstrated a greater COPVa-p at 5140m compared with sea level.

COPVm-l demonstrated minimal change between sea level and any of the three research altitudes. No significant differences were present in COPm-l; however during SRT with eyes open a *large* effect size demonstrated a greater COPVm-l at 3619 m compared with sea level (S3 Table). There was also no significant change in KJPS or SRT score between sea level and any of the three research altitudes (S4–S8 Tables). There were some *large* effect sizes demonstrated for some of the KJPS measures, but the differences were small and less than the smallest detectable difference for this technique [20].

Overall these results indicate there were few significant decrements in balance above 3619 m (Tables 1 and 2). COPVa-p during SRT with eyes open at 4600m was the single exception, and was *largely* reduced compared with 3619m (Table 2).

**Fig 3:**
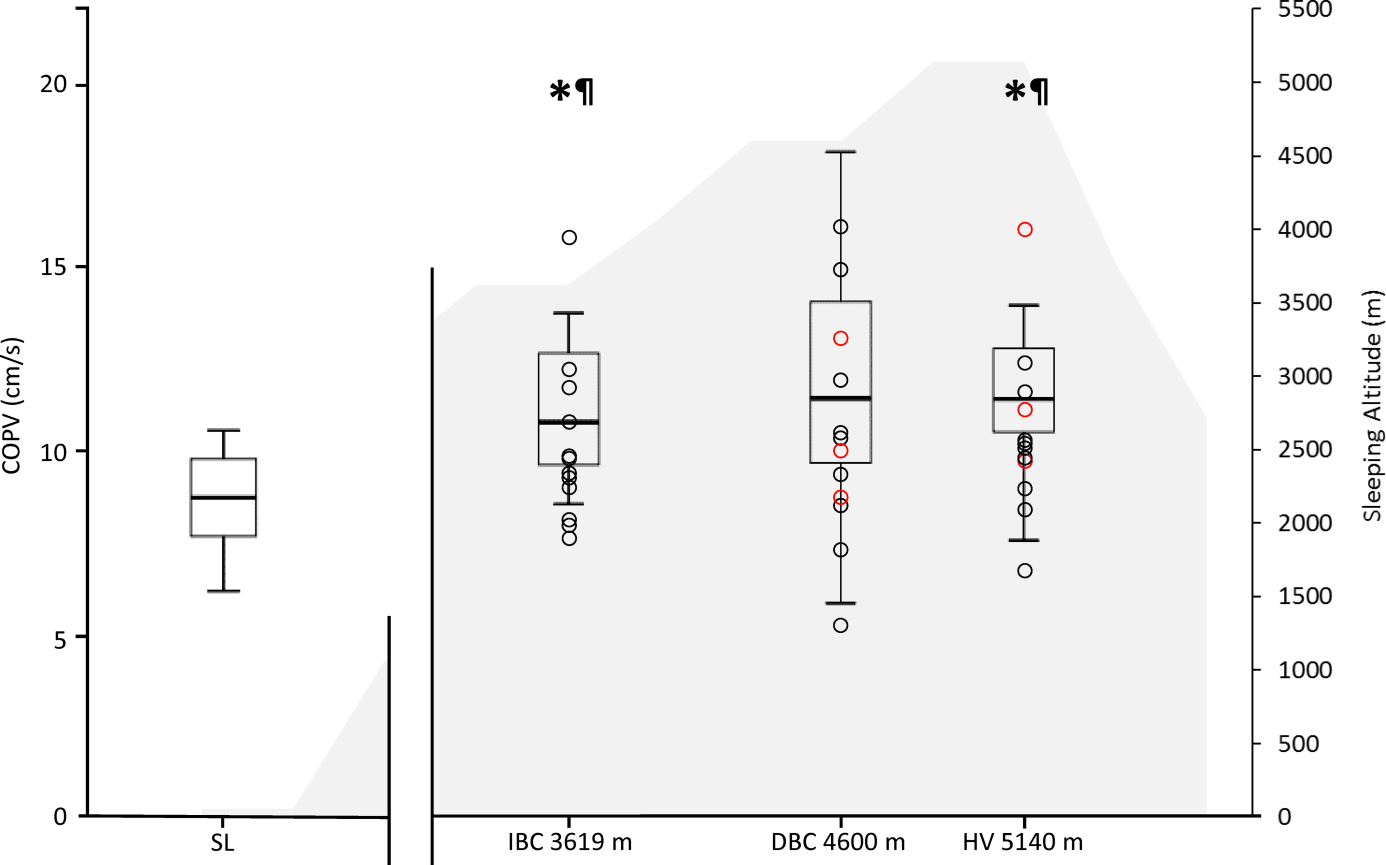
Centre of pressure total sway velocity (cm/s) for standing balance with eyes open along with altitude profile. Box plots show medians, quartiles, whiskers representing the max and min values, and dots representing individual participant values (O = positive LLS in 24 hrs before test). *p<0.05 vs. SL, ^**¶**^Cohen’s d > 0.8 compared with sea level.

As illustrated in Fig 3. the balance scores of participants with a positive LLS AMS score were distributed along the spectrum of balance scores presented by the entire group. SRT scores also failed to distinguish those with a positive AMS Score (S5 Table). In addition, there were no significant correlations between LLS Score or SpO_2_ and any postural sway variable (p>0.07; r<0.3).

## Discussion

This represents the first research study to investigate the effects of a prolonged gradual trekking ascent to high altitude on balance and knee joint position sense. The key findings were that total and anterior-posterior centre of pressure velocity were increased at 3619 m and above. Overall these impairments in postural control did not worsen at higher altitudes of 4600 m or 5140 m. In contrast, knee joint position sense scores remained stable throughout the trek. This is indicative of impairments in balance without impairments in KJPS at 3619 m and 5140 m when compared with sea level (113m). These findings demonstrate the importance for trekkers and mountaineers to be aware of continued balance impairments at altitudes of 3619m and higher. These impairments occurred despite a gradual ascent profile and may be used to inform guidance for future treks and expeditions.

The fact that balance was impaired at 3619 m and 5140 m when compared with sea level but the magnitude of impairment did not increase with altitudes above 3619 m (Table 1 and Table 2) is noteworthy. This lack of further balance impairment above 3619 m corroborate and supplement data from previous investigations [11,12], where posturographic parameters remained impaired over the course of a 4-day sojourn between 1630 m and 2590 m [11] and a 3-day stay at 4559 m [12]. Hypoxia or hypoxia-triggered events have been strongly linked with the occurrence of neurological deficits at high altitude [27]. Therefore, it is feasible to propose that decreases in arterial partial pressures of oxygen (P_a_O2) which occur during ascent to high altitude are the main cause of the accompanying impairment in postural control. However, the lack of relationship between SpO_2_ and postural sway in the current study and previous research [11] may indicate that the measures of pulse oximetry may not provide adequate sensitivity to detect these subtle changes in P_a_O2. It should be noted that the current study had a small sample for a correlational analysis. Considering the results of current and previous work [9,11,12], postural sway is shown to be impaired when SpO_2_ falls below 95%, but is likely to remain stable in relation to sea level when above 75%. Interestingly when military aircrew were acutely exposed over a 2-minute period to hypobaric hypoxia of 5486 m, 4267 m and 2438 m, decrements in postural sway were reported at both 4267 m and 5486 m compared to sea level, but no differences were reported between 4267 m and 5486 m [10]. In terms of the current study, it therefore seems unlikely that the acclimatisation of some body systems act to mitigate the decrements in postural control. Although further research is required to fully establish the mechanisms involved, it is more likely that inputs to the CNS from the somatosensory system are unaffected by the increased hypoxic hypoxemia and instead act to stabilise the impairments in visual and vestibular systems.

Consistent with previous studies that have reported that alterations mainly occur in total or anterior-posterior sway [7,10–12,23], the current study observed increases in total sway velocity (Table 1) and anterior-posterior sway velocity (Table 2). The current study found larger sway velocities and lower SRT balance scores with eyes closed than with eyes open (Table 1 and Table 2, S3 and S4 Tables), but sway velocities in the eyes open condition demonstrated larger impairments with altitude. This supports previous research at altitude where balance deterioration was larger in the eyes open condition [7,10,11]. Vision is purported to be the “first of the special senses to be altered by a lack of oxygen” [28]. The “zone of adaptation” from 3000 m– 5000 m is characterized by some visual impairment which is magnified at night or in the dark [28]. Above 5000 m is termed the “zone of inadequate compensation” [28] where participants have been reported to be unable to ignore inaccurate visual cues [13]. Excessive reliance on visual input is thought to be a natural compensatory strategy when afferent input from other nonvisual sources such as the vestibular system is reduced [29,30]. Therefore the decrements in postural control in the current study occurring at 3619 m but not deteriorating further are likely to be primarily due to impairments in the vestibular system which is exacerbated by slight impairment in the visual system. Targeted strategies may be implemented to offset the impairments to the vestibular and visual system and act to reduce decrements in static postural control. Virtual reality systems [31] and vision restricted rehabilitation [32] have been used effectively in to improve balance and gait stability in elderly patients [31]. These rehabilitation methods can teach the CNS to use or adjust the relative weight of other sensory inputs to substitute for the deficient vestibular system [31], or reverse the overreliance on the visual system [32]. Implementation of this form of prehabilitation prior to high altitude exposure may allow reduction in postural control impairments due to a more adaptable CNS.

Although participants in the current study demonstrated impairments in postural control during a prolonged altitude trek, there was no concomitant impairment in knee joint position sense. The current study is the first to indicate that the knee joint position sense is not affected by altitudes up to 5140 m and is not a contributory factor to the impairments in postural control. The control of joint position sense utilises specific afferent pathways conveying signals to the somatosensory cortex [33], and on exposure to hypoxia this brain region has been shown to maintain higher levels of oxygen delivery than the superior temporal gyrus which receives input from the vestibulocochlear nerve carrying information about balance [34]. Therefore, oxygen deficit greater than the levels that elicit impairments in postural control may be required to elicit impairments in joint position sense.

Since our study included fit military participants, the conclusions may not necessarily apply to older or sedentary individuals taking part in high altitude trekking activities. Although the segmented evaluation of postural control utilized in the current study allows for independent assessment of the visual, vestibular, and somatosensory systems, it does not evaluate their integration. The integration of these senses in maintaining postural control during a dynamic task has been previously assessed by computerized dynamic posturography (CDP) during rapid ascent at simulated altitude [7,13]. Future work should expand on the current study and the work of Wagner et al. [7,13] by investigating the weighted contribution of the postural control senses during gradual ascent to high altitude. This may involve the development of a field assessment similar to the CDP system which could be utilised during terrestrial expeditions. Alternatively the CDP system could be utilised at simulated altitude with a longitudinal gradual ascent profile. One final consideration for this future work is the acknowledgment of cognitive processes involved in dynamic postural control. The previously reported decrements in reaction time under hypoxia [35] are highly likely to impair dynamic postural control.

In conclusion, this is the first study investigating postural control and joint position sense over the course of a gradual ascent to high altitudes corresponding to an elevation profile of many commercial high altitude treks. Postural control was impaired at 3619 m and remained impaired without worsening with increasing altitude throughout the trek (up to 5140 m). Travelling over dangerous mountainous terrain wearing sometimes-heavy equipment has its own inherent risks and compounding this with impaired postural control makes the climber, trekker, or military operative particularly vulnerable to trips or falls [5]. Importantly, the findings of the current study may highlight the increased fall risk for individuals exposed to terrestrial altitudes between 3619 m and 5140 m even when utilising a gradual ascent profile. From a scientific perspective, the present findings suggest that trekking performance is mostly limited by impairments in postural control and not joint position sense which may be indicative of limitations within the vestibular system. Such findings should be considered during future trekking expeditions when considering specific strategies targeted at reducing or controlling such impairments.

## Supporting information

S1 Table SpO_2_ at different altitudes. (DOCX)

S2 Table Participant AMS occurrence at all altitudes. (DOCX)

S3 Table Centre of pressure velocity in the medial-lateral direction at different altitudes. (DOCX)

S4 Table SRT Scores at different altitudes. (DOCX)

S5 Table Number of subjects with abnormal and normal results for the Sharpened Romberg Test (SRT) and with or without acute mountain sickness (AMS)†. (DOCX)

S6 Table Relative error of knee joint position sense at different altitudes. (DOCX)

S7 Table Absolute error of knee joint position sense at different altitudes. (DOCX)

S8 Table Variable error of knee joint position sense at different altitudes. (DOCX)
