# Supplementary material for: Changes in balance and joint position sense during a 12-day high altitude trek: The British Services Dhaulagiri medical research expedition
Source: PLoS One. 2018 Jan 17;13(1):e0190919. doi: 10.1371/journal.pone.0190919 (PMC5771604; doi:10.1371/journal.pone.0190919)
Supplement: S1 Table — (DOCX) [file pone.0190919.s001.docx]

S1 Table. SpO_2_ at different altitudes

| Measurement | Day 1 1059 m | Day 7 IBC 3619 m | Day 10 DBC 4600 m | Day 12 HV 5140 m | P ANOVA Overall |
| --- | --- | --- | --- | --- | --- |
| Morning | 96.88 ± 0.84*^¶^ | 92.75 ± 1.58*^¶^ | 85.00 ± 4.47*^¶^ | 83.37 ± 4.21*^¶^ | <0.001 |
| Evening | 97.63 ± 0.52*^¶^ | 90.63 ± 5.40*^¶^ | 82.88 ± 3.04*^¶^ | 75.88 ± 5.17*^¶^ | <0.001 |

P ANOVA overall: Repeated Measures ANOVA within subjects effects (Trek Day 1, IBC, DBC, HV).

* p<0.05 compared to all other locations | ^¶^ Cohen’s d > 0.8 compared to all other locations
