# Supplementary material for: Changes in balance and joint position sense during a 12-day high altitude trek: The British Services Dhaulagiri medical research expedition
Source: PLoS One. 2018 Jan 17;13(1):e0190919. doi: 10.1371/journal.pone.0190919 (PMC5771604; doi:10.1371/journal.pone.0190919)
Supplement: S2 Table — (DOCX) [file pone.0190919.s002.docx]

S2 Table. Participant AMS occurrence at all altitudes.

| Participant | 1 | 2 | 3 | 4 | 5 | 6 | 7 | 8 | 9 | 10 | 11 | 12 |
| --- | --- | --- | --- | --- | --- | --- | --- | --- | --- | --- | --- | --- |
| 1059 m AM | - | - | - | - | - | - | - | - | - | - | - | - |
| 1456 m PM | - | - | - | - | - | - | - | - | - | - | - | - |
| 1456 m AM | - | - | - | - | - | - | - | - | - | - | - | - |
| 2017 m PM | - | - | - | - | - | - | - | - | - | - | - | - |
| 2017 m AM | - | - | - | - | - | - | - | - | - | - | - | - |
| 2430 m PM | - | - | - | - | - | - | - | - | - | - | - | - |
| 2430 m AM | - | - | - | - | - | - | - | - | - | - | - | - |
| 3107 m PM | - | - | - | - | - | - | - | - | - | - | - | - |
| 3107 m AM | - | - | - | - | - | - | - | - | - | - | - | - |
| 3619 m PM | - | - | - | - | - | - | - | - | - | - | - | - |
| 3619 m AM | - | - | - | - | - | - | - | - | - | - | - | - |
| 3619 m PM | - | - | - | - | - | - | - | - | - | - | - | - |
| 3619 m AM | - | - | - | - | - | - | - | - | - | - | - | - |
| 4072 m PM | AMS | AMS | - | AMS | - | - | - | - | - | - | - | - |
| 4072 m AM | AMS | - | - | - | - | - | - | - | - | - | - | - |
| 4600 m PM | - | - | - | AMS | - | - | - | - | AMS | - | - | - |
| 4600 m AM | S-AMS | - | - | - | - | - | - | - | - | - | AMS | - |
| 4600 m PM | - | - | - | - | - | - | - | - | - | - | - | - |
| 4600 m AM | - | - | - | - | - | - | - | - | - | - | - | - |
| 5140 m PM | - | - | - | AMS | - | - | AMS | - | - | - | - | - |
| 5140 m AM | AMS | AMS | - | - | - | - | - | - | - | - | - | - |
| 5140 m PM | AMS | - | - | - | - | - | - | - | - | - | - | - |

AMS = LLS score ≥3 in the presence of a headache | S-AMS = LLS score ≥6
