# Supplementary material for: Changes in balance and joint position sense during a 12-day high altitude trek: The British Services Dhaulagiri medical research expedition
Source: PLoS One. 2018 Jan 17;13(1):e0190919. doi: 10.1371/journal.pone.0190919 (PMC5771604; doi:10.1371/journal.pone.0190919)
Supplement: S3 Table — (DOCX) [file pone.0190919.s003.docx]

S3 Table. Centre of pressure velocity in the medial-lateral direction at different altitudes

| Measurement | Eyes | Sea level | IBC 3619 m | DBC 4600 m | HV 5140 m | P ANOVA Overall |
| --- | --- | --- | --- | --- | --- | --- |
| Standing Balance | Open | 4.89 ± 0.92 | 5.39 ± 1.44 | 5.51 ± 1.56 | 5.18 ± 1.27 | 0.193 |
| Standing Balance | Closed | 6.10 ± 1.40 | 6.08 ± 1.71 | 6.67 ± 1.85 | 5.92 ± 1.44 | 0.185 |
| SRT | Open | 16.88 ± 7.08 | 26.56 ± 9.27**^¶^** | 18.75 ± 4.83 | 22.33 ± 7.03 | **0.011** |
| SRT | Closed | 38.37 ± 20.67 | 33.52 ± 9.85 | 43.42 ± 17.84 | 43.48 ± 24.43 | 0.254 |

Data are presented as mean COPVm-l in cm/s ± standard deviation
P ANOVA overall: Repeated Measures ANOVA within subject effects (SL, IBC, DBC, HV).
^¶^ Cohen’s d > 0.8 compared with sea level
