# Supplementary material for: Changes in balance and joint position sense during a 12-day high altitude trek: The British Services Dhaulagiri medical research expedition
Source: PLoS One. 2018 Jan 17;13(1):e0190919. doi: 10.1371/journal.pone.0190919 (PMC5771604; doi:10.1371/journal.pone.0190919)
Supplement: S4 Table — (DOCX) [file pone.0190919.s004.docx]

S4 Table. SRT Scores at different altitudes

| Measurement | Eyes | Sea level | IBC 3619 m | DBC 4600 m | HV 5140 m | P ANOVA Overall |
| --- | --- | --- | --- | --- | --- | --- |
| SRT | Open | 240.0 ± 0.0 | 234.5 ± 18.1 | 240.0 ± 0.0 | 240.0 ± 0.0 | 0.343 |
| SRT | Closed | 194.6 ± 64.8 | 227.1 ± 22.5 | 216.8 ± 48.9 | 207.2 ± 58.6 | 0.378 |

Data are presented as average score in s ± standard deviation
P ANOVA overall: Repeated Measures ANOVA within subject effects (SL, IBC, DBC, HV).
