# Supplementary material for: Changes in balance and joint position sense during a 12-day high altitude trek: The British Services Dhaulagiri medical research expedition
Source: PLoS One. 2018 Jan 17;13(1):e0190919. doi: 10.1371/journal.pone.0190919 (PMC5771604; doi:10.1371/journal.pone.0190919)
Supplement: S5 Table — (DOCX) [file pone.0190919.s005.docx]

S5 Table. Number of subjects with abnormal and normal results for the Sharpened Romberg Test (SRT) and with or without acute mountain sickness (AMS)†

| Altitude | SRT | | | | |  | |  |  |  |
| --- | --- | --- | --- | --- | --- | --- | --- | --- | --- | --- |
|  |  | | Scores (s) | | | AMS (# participants) | | | | |
|  |  |  | | Median | SD | With | Without | | | |
| 3619 m | Abnormal | 3 | | 187.3 | 9.6 | 0 | 3 | | | |
|  | Normal | 8 | |  |  | 0 | 8 | | | |
| 4600 m | Abnormal | 3 | | 181.3 | 66.9 | 1 | 2 | | | |
|  | Normal | 9 | |  |  | 1 | 8 | | | |
| 5140 m | Abnormal | 5 | | 183.0 | 67.5 | 0 | 5 | | | |
|  | Normal | 7 | |  |  | 2 | 5 | | | |

†A normal SRT is 240 seconds. Acute mountain sickness was scored using the Lake Louise self-completed questionnaire on the same morning of the SRT. LLS score ≥3 in the presence of a headache was used to diagnose AMS.
