# Supplementary material for: Changes in balance and joint position sense during a 12-day high altitude trek: The British Services Dhaulagiri medical research expedition
Source: PLoS One. 2018 Jan 17;13(1):e0190919. doi: 10.1371/journal.pone.0190919 (PMC5771604; doi:10.1371/journal.pone.0190919)
Supplement: S6 Table — (DOCX) [file pone.0190919.s006.docx]

S6 Table. Relative error of knee joint position sense at different altitudes

| Measurement | Sea level | IBC 3619 m | DBC 4600 m | HV 5140 m | P ANOVA Overall |
| --- | --- | --- | --- | --- | --- |
| 10-30° Flexion | 0.56 ± 2.78 | -3.36 ± 4.63 | -0.19 ± 3.60 | -1.48 ± 3.86 | **0.011** |
| 30-60° Flexion | 1.72 ± 2.78 | 0.21 ± 1.81 | 1.15 ± 2.48 | -0.45 ± 3.35 | 0.457 |

Data are presented as mean relative error in ° ± standard deviation
P ANOVA overall: Repeated Measures ANOVA within subject effects (SL, IBC, DBC, HV).
The 60-90° measurement was not recorded at DBC
