# Supplementary material for: Changes in balance and joint position sense during a 12-day high altitude trek: The British Services Dhaulagiri medical research expedition
Source: PLoS One. 2018 Jan 17;13(1):e0190919. doi: 10.1371/journal.pone.0190919 (PMC5771604; doi:10.1371/journal.pone.0190919)
Supplement: S7 Table — (DOCX) [file pone.0190919.s007.docx]

S7 Table. Absolute error of knee joint position sense at different altitudes

| Measurement | Sea level | IBC 3619 m | DBC 4600 m | HV 5140 m | P ANOVA Overall |
| --- | --- | --- | --- | --- | --- |
| 10-30° Flexion | 2.67 ± 1.43 | 4.92 ± 2.73**^¶^** | 3.03 ± 1.86 | 3.18 ± 2.60 | 0.073 |
| 30-60° Flexion | 2.78 ± 1.87 | 2.33 ± 1.20 | 2.48 ± 1.56 | 2.64 ± 2.02 | 0.734 |
| 60-90° Flexion | 3.06 ± 1.54 | 2.78 ± 2.39 |  | 3.50 ± 3.54 | 0.537 |

Data are presented as mean relative error in ° ± standard deviation
P ANOVA overall: Repeated Measures ANOVA within subject effects (SL, IBC, DBC, HV).

^¶^ Cohen’s d > 0.8 compared with sea level

The 60-90° measurement was not recorded at DBC
