# Supplementary material for: Changes in balance and joint position sense during a 12-day high altitude trek: The British Services Dhaulagiri medical research expedition
Source: PLoS One. 2018 Jan 17;13(1):e0190919. doi: 10.1371/journal.pone.0190919 (PMC5771604; doi:10.1371/journal.pone.0190919)
Supplement: S8 Table — (DOCX) [file pone.0190919.s008.docx]

S8 Table. Variable error of knee joint position sense at different altitudes

| Measurement | Sea level | IBC 3619 m | DBC 4600 m | HV 5140 m | P ANOVA Overall |
| --- | --- | --- | --- | --- | --- |
| 10-30° Flexion | 2.08 ± 1.26 | 1.79 ± 1.35 | 1.59 ± 1.10 | 1.93 ± 1.44 | 0.809 |
| 30-60° Flexion | 2.04 ± 1.31 | 2.46 ± 1.61 | 1.78 ± 0.85 | 1.12 ± 0.77**^¶^** | 0.123 |
| 60-90° Flexion | 1.82 ± 0.66 | 1.48 ± 0.79 |  | 1.90 ± 1.05 | 0.314 |

Data are presented as mean relative error in ° ± standard deviation
P ANOVA overall: Repeated Measures ANOVA within subject effects (SL, IBC, DBC, HV).

^¶^ Cohen’s d > 0.8 compared with sea level

The 60-90° measurement was not recorded at DBC
